# Hospital and Institutional News

**Published:** 1914-04-25

**Authors:** 


### HOSPITAL AND INSTITUTIONAL NEWS.

### THE KING AND A PARIS HOSPITAL.

As we go to press this week it is too soon to learn whether or not the King has been able to find time to visit the English Hospital in Paris during his necessarily brief and crowded stay in the French capital. The great welcome accorded to him and to the Queen by the French people, the brilliant weather, and the genuine enthusiasm displayed, seem to give a rebirth to—or shall we say to baptise and confirm?—the *Entente* associated with the reign of King Edward. The national character expressing itself in the mutual co-operation of the two nations is one of which the voluntary hospital system is our proudest achievement, and nothing could better display this jewel among England's enterprises than the King's visit, if he can find time to make it, to the Hertford British Hospital. Founded in 1871, and reopened almost to a month thirteen years ago, the English Hospital in Paris is an institution with forty beds, situated in the Rue de Villiers-Levallois-Perret. Its president is the Hon. L. D. Carnegie, M.V.O., and five members are appointed by Government. The staff consists of three physicians, two surgeons, one house surgeon, apart from specialists, the matron, Miss Morton, and nine nurses. The secretary is Mr. Frank W. Wicker, and there were nearly 300 in-patients last year.

### THE DOCTORS' WIVES ASSOCIATION.

It is too early to enable us in the present issue to state definitely the procedure which will be followed in organising the Doctors' Wives Association, the scheme of which was fully explained in the Supplement contained in The Hospital for March 21, 1914. We are gratified to find that the scheme has commended itself to some of the most representative and respected members and leaders of the medical profession and their wives. It will rest with those who have signed and sent in the coupon which we published in The Hospital of 21st ultimo and subsequent issues to determine exactly what course shall be followed in the initial stages of the work contemplated by this important Association. We are grateful to those who have shown their confidence by sending in their names, and we have determined to keep the list open for a fortnight longer to enable any who wish to join to sign the coupon on page 108 and forward it to "Co-operation," c/o The Scientific Press, 28 Southampton Street, Strand, London, W.C., without fail. One point which gives us the greatest satisfaction is the quality and representative character of those who have joined the Association initially, which demonstrates that the proposals are considered of value and that they will in fact fulfil the requirements and further the best interests of the wives of practitioners who join this Association.

### FUNERAL OF MR. SYDNEY PHILLIPS.

After a prolonged period of illness, the death has occurred, and the funeral taken place, of Mr. Sydney Phillips, steward of St. Thomas's Hospital. Mr. Phillips was forty-five years of age, and had served St. Thomas's Hospital for fifteen years, and before that he had held a post at St. Mary's. He was a most valuable officer of a type which St. Thomas's and the London institutional world can ill afford to lose. He had taken great pains to qualify himself for a high administrative position, more especially by graduating at London University, and by becoming a barrister. He leaves a widow and two sons, to whom we cannot refrain from offering a note of personal sympathy, since Mr. Phillips was perhaps most characteristically a busy man in this, that he could always find time to do something for other people—and ourselves, among many others, have a vivid personal memory and a lively gratitude for his co-operation and assistance on several occasions when his ability was invoked. All our readers will turn with interest to the special memoir, contributed by one with unrivalled opportunities for appreciating his character and work, which appears on page 104. The illustration which accompanies the article has been made from a favourite photograph, which those who knew him best have courteously allowed us to reproduce, and is the best available pictorial record of his appearance and engaging address.

### ADMINISTRATIVE PROMOTIONS IN SANATORIA.

Mr. F. P. Carroll, assistant secretary to Charing Cross Hospital, has been appointed steward at King Edward VII. Sanatorium, Midhurst, a post vacated by Mr. W. H. Booth on his appointment to the secretaryship of the Jessop Hospital for Women, Sheffield. Mr. Carroll is known to our readers as a contributor to The Hospital, especially on practical points—such as those dealt with in the *Institutional Worker* Supplement—of which his article on "Water-Softening for Institutions: The Type and Cost of Various Appliances Compared," which appeared in our issue of June 22, 1912, is an example. He was also known to his colleagues as an active spirit on the *Gazette*, where his journalistic instincts were often recognisable, and had the result of signing apparently anonymous articles with very characteristic flourishes between the lines. Mr. Booth, whom he succeeds at Midhurst, was formerly steward at the London Open-air Sanatoria, Wokingham, and previously was in the secretarial office at the Royal National Hospital, Ventnor.

### EFFECT OF SPECIALISM ON INFIRMARY WORK.

For some time past the Bristol Guardians, as noted in The Hospital, have had under consideration a scheme for the reorganisation of the provision made for the institutional relief of their poor. They have now decided to specialise the three institutions they possess. The Southmead Infirmary is to receive all the acute sick cases. When completed it will comprise five blocks, with seventy-two beds in each, a maternity ward of twenty beds, a children's block of fifty-four beds, wards for sixty-four convalescent cases, and a male consumptive ward of thirty beds, making a total of 528 beds. The Stapleton Workhouse is to be used for imbeciles and chronic senile cases not requiring infirmary treatment. It will have accommodation for at least 530 inmates. The Eastville Workhouse is to be used for healthy inmates, and will have about 1,000 beds, including a nursery for children under three. The medical staff is to consist of a medical superintendent, two assistant medical officers, a consulting surgeon, and a consulting physician. The medical superintendent and an assistant medical officer are to be resident at the Southmead Infirmary, the other assistant medical officer is to reside at the Stapleton Workhouse.

### TWO CRITICISMS OF THE BRISTOL SCHEME.

This decentralised staff is also to carry out the ordinary medical work at Eastville, with the assistance of a medical practitioner resident near the workhouse, who is to be called in only in cases of urgency. In addition, the medical staff is to have charge of the children's homes. The first criticism that suggests itself is that the proposed medical staff is obviously inadequate for the work assigned to it. The second criticism is that the system of a visiting surgeon and physician does not meet present-day requirements. The increase in the specialisation of medical work has rendered it obsolete. A much more elaborate arrangement is needed nowadays. Probably the best system for a Poor-Law infirmary is to appoint as medical superintendent a man skilled in general surgery, and to give him power to call in specialists in other departments as required. In this way he can obtain the assistance, when necessary, of an ophthalmic surgeon, a laryngologist, an *x*-ray expert, a neurologist, and so on. Where experience shows that the number of cases justifies it, any one or more of these experts can be appointed as visiting medical officers. In this way, and in this way only, can the work in a Poor-Law infirmary be brought into line with the standard achieved at our voluntary hospitals.

### A SCARE FOR HOSPITAL BIBLIOGRAPHERS.

A statement is made disquieting to all who are interested in medical bibliography to the effect that the Library of the Surgeon-General, Washington, should be transferred and become part of the Library of Congress. It appears that an amendment to the Army Appropriation Bill passed the Senate on March 28 without consultation, as is said, with the Surgeon-General of the Army or the head of the Library of Congress. Complaint is rightly made, and the matter is commented upon adversely as being hasty and unwise legislation, without justification or reason; further, it is said that the Library of Congress has no room for it and that the Librarian of Congress does not want it. There can indeed be but few who know anything of the literature of hospitals and nursing who have not heard of that monumental work, the "Index Catalogue," the compilation of which represents so important an item in the work of the Library of the Surgeon-General. This wonderful Index was first issued in the year 1880, and completed its first series, A to Z, in 1895. The issue of the second series commenced in 1896, and has now reached the letter T, it being usually issued at intervals of a year between each volume. In all thirty-four volumes have now appeared, the latest not having yet reached this country. The Index is printed in all languages, and is an exhaustive catalogue of authors and subjects in every branch of literature connected with hospitals, medicine, surgery, and nursing. So great a friend has the Index proved to all who have frequently to turn to its pages that any news which savours of impairment of its usefulness would cause widespread regret in the hospital and medical world.

### DOCTORS, DISEASE, AND THE LAW OF LIBEL.

Sir Victor Horsley made good use of the opportunity afforded to him when giving evidence at the twenty-ninth meeting of the Royal Commission on Venereal Diseases by calling attention to the condition of the nation's vital statistics, and by criticising the present system of registration, while detailing a reform of the Registrar's Office, including the confidential certification of death which was mooted some years ago. In addition, he laid stress on the question of medical men and the law of libel. It was obvious, he said, that from the point of view of public health a medical practitioner should be able to warn anyone, who, to his knowledge, was likely to be infected with venereal disease, but as the law stood at present a medical man so doing would render himself libel to an action for libel which might easily result in his ruin. A practitioner ought to be free to state his opinion as to the cause of a particular disease, provided that such information should be given only to those who had a right to ask for it. Sir Victor, who favoured instruction of children in sex matters, provided that Nature-study alone was given in class, and that the questions relating to human reproductive systems were given individually alone, also spoke in favour of notification, and said that the resentment such a proposal would probably meet with, both from the profession and the public, would, he thought, prove as short-lived as that which had originally greeted the notification of scarlet fever. The notification should be confidential. He supported treatment in special wards at general hospitals, and urged that every health authority should have its own bacteriological laboratory for diagnosis, adequate facilities for which could only be provided in his opinion by subsidies from the State. Dr. Florence Willey, assistant physician for diseases of women at the Royal Free Hospital, opposed notification, and advocated the indentification of all material sent for diagnosis solely by number. Whatever opinion individuals may hold of Sir Victor Horsley's suggestions, he deserves great credit for having made a more thorough attempt than many other witnesses to cover the whole ground.

### A NEW GENERAL HOSPITAL FOR LINCOLNSHIRE.

A meeting has been held at Scunthorpe, Lincolnshire, at which a provisional committee, representative of the professions and interests of the district, was formed to prepare a scheme for a new hospital, both for accident and general purposes. The meeting was presided over by the Rev. C. T. Rust, who is chairman of the existing cottage hospital at Frodingham, and the convener of the meeting, Mr. G. H. Stampe, explained that the proposal arose out of a circular sent by one of the local firms urging increased subscriptions to the cottage hospital, and out of the simultaneous proposal of the trade union leaders that a new general hospital should be built. The cottage hospital, he added, had done good service, but the limits of its capacity had been reached. The provisional committee will soon meet, it is hoped, to decide on the scope and general features of the new hospital.

### "AN INSANITARY AND OBSCURE VILLAGE."

A very interesting and rather extensive scheme, writes a correspondent, for cottages in Aran for the betterment of the inhabitants of this insanitary and obscure village is about to take place. The Congested Districts Board has stipulated to provide sites and give plots of one acre each. The poor fishermen hitherto possessed no garden, and the old houses were built on a sort of back-to-back plan, where fever frequently visited the homes. The minimum cost of each cottage will be about £90. The Board state that there will be no added expense, as they will provide engineering supervising, as well as ground. A history of the difficulties connected with these grants, our informant continues, is to be found in the life of Father Farragher, who has laboriously struggled to secure the land on which these houses are to be erected by these poor families of Killeany Village, Aran. Such as he, rather than the politicians, are the true Irish patriots.

### BATHS FOR CONTINUOUS IMMERSION.

Every post brings to the Editor's table a number of inquiries on practical points, showing the need and value of the *Institutional Worker* section. We want to enlarge it and make it useful to each worker, in whatever department of a hospital or institution he or she may be employed. The co-operation of all such workers and of the responsible officers of the institutions generally is therefore invited to enable this department of the paper to attain its maximum usefulness. Will the more practical of our readers keep an eye on this section and help us with a little personal service by making it known to their staff? The columns for Questions Invited and Questions To Be Answered present an opportunity for co-operation which, if availed of, may result in many advantages to the individual. In particular we would direct the attention of medical superintendents and others to the important question which appears this week on page 2 of the *Institutional Worker*. Such a bath as is there referred to is but little known, and our readers are invited to give their experience.

### THE HEN OUT-PATIENT.

The recent treatment of a hen at the out-patient department of the Bolingbroke Hospital, to which it was brought with a broken leg by a small boy, raises more than one interesting point, especially for almoners. Was its reception by the kind house surgeon an instance of hospital abuse? was the boy who had saved up his money, it appears, to purchase the hen, in *loco parentis* and responsible for contributing towards its treatment? or, if not, were the boy's relatives to be held responsible? Failing them, was the hen really a case ineligible for treatment, which ought to have been sent on to the Poor-Law infirmary? Let no institutional officer, whether a lady almoner or not, take a contemptuous view of these questions, nor attempt to cover intellectual laziness over his profession by pooh-poohing them as unworthy of his or her valuable time. There is no better way of testing rules and regulations than in their application to an unexpected case. Brought to this test, we can very soon place our finger on a weak spot, if one exists. For example, supposing the original idea which induced some of the hospitals to adopt the modern almoner system, namely, its value in preventing "hospital abuse," were still the mainspring of all almoners' work, and that the constructive, after-care side were still regarded as a dangerous fad, likely to "pauperise" the out-patients subjected to it, and to involve the hospital in trouble and expense for which it would have no adequate result to show—the advent of this hen at such an out-patient department would have emphasised two obvious but useful things.

### THE HEN AND HOSPITAL ECONOMY.

First, it is clear that aid must be rendered, abuse or no abuse, to casualty cases even if, as in the case of the hen, there is no doubt of the ineligibility of the patient if the letter of the regulations is to be observed; and, secondly, that humanity and considered help for patients, however troublesome in themselves, and indeed irregular, are still more economical in the long run than any mere saving system. Economy is the art of spending wisely, one of the results of which is a saving of expense; and the true nature of hospital economy could hardly be shown better than in the case of this hen, whose reception, treatment, and instructions to call again will give the Bolingbroke Hospital an advertisement locally and generally which will rally all the sympathies of the neighbourhood to its support, and (who knows?) lead to a regular batch of fresh subscriptions or legacies. The casualty officer was not thinking of these things when he decided to treat the hen as if, in the language of St. Francis, she were his "little sister." He was following a humane impulse merely. Such impulses are the mainspring of the voluntary hospital system, and should be also the inspiration of hospital management and finance.

### OPERATIVE WORK IN A LONDON INFIRMARY.

It was anticipated in many quarters that with the advent of the National Insurance Act there would be a diminution in the number of patients in Poor-Law infirmaries, but, as we have shown, this has generally proved to be incorrect. Few districts have been more affected by the Act than Bermondsey, where the population mainly consists of waterside labourers and people dependent on casual labour. Dr. Bell, the medical superintendent, states, in his annual report, that the number of cases treated in the wards during the year amounted to 3,366, an increase of 205, as compared with last year. These comprised both acute and chronic diseases, but the ratio of the former to the latter had somewhat increased. The number of cases cured or relieved who were transferred to other establishments of the Guardians, or who took their own discharge, amounted to 2,245, or 162 in excess of the previous year. Operations, excluding catheterisations and the like, numbered 430. Dr. Bell records his thanks to his colleagues, Dr. Crean and Dr. Eccles, for their work, and especially with regard to surgical operations. He also acknowledged the good services of Mr. W. Burke, the dispenser, both for his accurate work and the economy he has effected in his department. To be able to add, as he does, that the "entire administrative and nursing staff has worked with good will and with the minimum of friction" is a tribute alike to his subordinates and to himself.

### ISOLATION HOSPITAL OFFICER VINDICATED.

As a result of complaints made by Dr. James Souter and Mr. Willmann against the administration of the Wimbledon Isolation Hospital, in which Dr. Clapham, the hospital's medical officer, was aspersed, the Public Health Committee of the Borough of Wimbledon approached the Local Government Board. A letter from the Board has since been received which referred to the reports of the medical officer, and went on to say that in the light of these it did not propose to take any further action in the matter. The Public Health Committee have, therefore, passed a resolution to the effect that the committee is satisfied that the reflections cast upon Dr. Clapham and his administration were without foundation. Dr. Clapham will receive congratulation for this vindication, if only on the general ground that while complaints made against institutional officers are, taken as a whole, few and far between, they are, when made, from the nature of the case, of a sensational and damaging character. A resolution of vindication passed by a Public Health Committee is, therefore, the only satisfactory way, from the point of view of those implicated by such charges, for the incident to be not only closed but prevented from resurrecting. As The Hospital noted at the time, the complaints were concerned with cross-infection.

### A FIRE IN A DOCTOR'S SURGERY.

A Battersea practitioner had an unpleasant experience last Saturday, when a strong smell of gas induced him to enter one of the lower rooms of his house, whereupon an explosion occurred so forcibly as to blow out all the front windows and to work considerable havoc in several rooms. Mr. Clanchy, the practitioner in question, was himself thrown to the ground, burnt in several places, much bruised and injured, and had to be conveyed to St. Thomas's Home suffering from shock. He is understood to be progressing, and luckily, owing to the prompt assistance given by the Fire Brigade, the fire, which unchecked would have been very serious, was quickly put out. We hope, however, that a careful investigation of the cause of the explosion will be made, partly because even simple precautions are too often neglected, and, consequently, great risks are run not only by private individuals, who may be given the unflattering excuse of not knowing any better, but even by institutions themselves. One reason of this is undoubtedly the strange belief that prevails so widely among administrative staffs that the how and why of a fire are matters to be concealed as much as possible as reflecting upon their watchfulness or ability. The truth is that nothing can reflect upon them more than this very secretiveness. Probably someone has been unwatchful or careless; but it is exactly unwatchfulness and carelessness which have to be guarded against, and the only way to do this is to collect every available instance of how and when these general defects of human nature have arisen or are likely to arise.

### A CHAPLAINCY DEPARTURE AT GREENWICH.

Since the death of the Rev. S. Martyn Bardsley, late Vicar of Greenwich, the committee of the Seamen's Hospital have entered into an arrangement with the Missions to Seamen for the performance of the duties of chaplain at the *Dreadnought* Hospital. The Missions have arranged for the Rev. J. A. Bishop, one of their chaplains, to reside in the neighbourhood of the hospital. The wards are also visited occasionally by the Rev. N. Lash, the superintendent, and other clergy connected with the Missions. The principal advantage of this arrangement is that among the inmates of the hospital are frequently found patients well known to the clergy of the Missions to Seamen, and it is hoped that this new departure will be of great benefit to the sick and injured seamen who come to the *Dreadnought*. The change is also of general interest in that it indicates how much may be gained by applying to this department the same energy, care, and enthusiasm that is expected in all others. Too often in the past hospital chaplaincies have been the not too much desired, and generally not too valuable, perquisites of a particular incumbency. Here we see the importance of giving these appointments on the ground of qualification alone.

### THE DOG AND RESEARCH.

The Dogs Protection Bill, the object of which is to prohibit the performance of vivisectional experiments on dogs, has elicited a memorial to the Home Secretary protesting that grave injury would result to research were the Bill passed, which has been signed by a variety of institutional and medical men. We append below the names of some of those hospital administrators who have taken this view: Lord Knutsford, chairman of the London Hospital; Sir Joseph Fayrer, superintendent of the Edinburgh Royal Infirmary; Sir Frederick Macmillan, chairman of the National Hospital for Paralysed and Epileptic, Queen Square; Mr. J. T. Helby, chairman of the Metropolitan Asylums Board; Dr. Edward Goodall, superintendent of the Eastern Hospital; and Lord Northbrook, president of the Cancer Hospital. The Presidents of the Royal Colleges, Sir William Osler, and many others also signed.

### THE INSURANCE ACT AND INFIRMARY CONFINEMENTS.

It is apparent that the provisions of the National Insurance Act so far as they relate to confinements in Poor-Law infirmaries have not yet been fully recognised by Boards of Guardians. The Local Government Board have addressed a letter to the Southwark Board of Guardians on the question. It appears that a woman, whose husband had been out of work for a long period, was compelled to be confined in the Guardians' institution. When she came out, she asked the doctor to sign the maternity form, as she could do with the money to clothe the child with. But the doctor declined to do so, saying that the Guardians would not permit it, as she was not entitled to the maternity benefit. The Insurance card was fully paid up. The Local Government Board state that the powers and the duties of the Guardians in regard to the recovery of the cost of maintenance from any person legally liable for the same are not affected by any of the provisions of the National Insurance Act. But, having regard to Section 3 of the Act, which renders void any agreement to assign benefits, the Board are of opinion that the Guardians should not endeavour to intercept any benefit under the Act, and should not make use of the machinery and requirements of the Act to obtain the money payable as benefit or any part of it.

### INFIRMARIES AND THE ABUSE OF BENEFITS.

The Guardians have referred the matter to a committee for consideration. It is a serious position, so far as the outside ratepayer is involved, and is another instance of overlapping, whereby the ratepayer is called upon to bear the brunt. The Old-Age Pensions have created trouble in a similar manner. Some recipients of the pension have lived in the workhouses for weeks, allowing the five shillings to accumulate. These have then taken their discharge, and in a few days have spent the whole of the pension money in drink, and as soon as it is gone returned to the workhouse, there to remain at the ratepayers' expense until the pension has once more accumulated. The benefits of the National Insurance Act are to be unchecked in a similar way, according to the letter from the Local Government Board. Men, entitled to benefits, can come into the Poor-Law institutions and receive treatment, whilst outside their sick money accumulates. When they are discharged they can draw the full amount, which they can spend as they like. The whole difficulty is that while such procedure is an abuse of public money and public institutions, its rigorous repression might lead to there being no benefit at all, for to hand over the benefit money to institutions or private practitioners, unless also better treatment could be guaranteed, would be as much an imposition on the insured as the abuse of the benefit by some of them is an imposition on the ratepayers and the infirmaries.

### A BEQUEST TO SANATORIUM CONVALESCENTS.

Mr. Henry Kirkpatrick, J.P., of Tyldesley, Lancs, cotton spinner, has left £500 upon trust to pay the annual income therefrom to such of the inhabitants of the urban district of Tyldesley with Shakerley, irrespective of party, sect, or creed, as may have been confined in a sanatorium, hospital, or infirmary, and require a change of air and rest. It will be noted that this bequest differs not only from those which are given to change-of-air funds aiming to give country holidays to slum-dwellers, but also from the well-known funds for providing convalescent homes for hospital patients. It would seem in the present case that the testator had in his mind a desire to begin almost where the institutional convalescent home leaves off, or at any rate to supply the means whereby ex-patients can get back to health removed from the institutional atmosphere. Whether this implies a criticism in the giver's mind of the efficacy of institutional convalescent treatment as a whole we do not know. What are our readers' opinions?

### THE MEDICAL STAFF OF THE CITY OF WESTMINSTER INFIRMARY

One result of the formation of the City of Westminster Union has been to increase the number of inmates in the Fulham Road Infirmary of that Union. The proposed closing of the Edmonton Workhouse of the Union will undoubtedly lead to a still further increase. Under these circumstances, the Guardians have been considering the numerical strength of the medical staff of the infirmary, and have decided to appoint an additional medical officer. The medical staff with this addition will consist of a medical superintendent, with three assistant medical officers. This staff, however, cannot be considered sufficient for the work. The certified accommodation of the infirmary is 776 beds; that of the adjacent workhouse, which is attended by the medical staff of the infirmary, is 2,111 beds. At the present time there are 619 inmates in the infirmary and 1,437 in the workhouse. The minimum medical staff for the working of two institutions of this size, having regard to the concentration of work resulting from the amalgamation of parishes to form the new Westminster Union, should be a medical superintendent with five assistant medical officers. A small medical staff means that the work is not properly done, and that patients who should be cured and discharged accumulate in the infirmary. That something of this kind has occurred in the past owing to the understaffing of the infirmary is shown by the fact that in 1912, with 2,764 admissions in the year, the average number of occupied beds was 562. This gives an average of five patients annually to each occupied bed—a number considerably below what should be the case if the medical work were being efficiently performed.

### CHRISTIANITY AND MEDICINE IN CHINA.

The commemoration of the two hundred and thirteenth anniversary of the Society for the Propagation of the Gospel was made the occasion, among other things, of drawing attention to the relation between medicine and Christianity in China. It was pointed out that though there were an average of only two qualified medical practitioners to half a million persons—which sounds like the Chinese version of our panel practice at home!—yet, from the missionary point of view, the medical power to relieve pain in the hands of the medical missionaries gave Christianity an influence, especially over superstitious persons, which no mere preaching could give. Medical missions, it was stated, were among the most powerful agents of the changes now fermenting in China, and Chinese statesmen were very alive to the need for making use of missionaries in medical education. It seems, in fact, that Chinese medical students tend to become Christians, and that the medical missionary is, as it were, a man doubly armed to drive European notions into the venerable cradle and parent of civilisation, which, whether regretted or unregretted, whether or no it passes away, China will always be in the mind of the artist, the philosopher, and the medical historian.

### WHALE OIL AS A NEW FOOD.

At an evening meeting of the Pharmaceutical Society, held at 17 Bloomsbury Square, London, on Tuesday, Professor Arthur W. Crossley, F.R.S., read a paper on the "Preparation and Commercial Uses of Hydrogen," in which he drew attention to the fact that whale oil and cotton-seed oil are now being rendered capable of utilisation as food-stuffs by treating the oils with hydrogen. Hydrogen is passed through the heated oils in presence of a calalytic agent, usually metallic nickel, and a hard white substance is produced, which is practically odourless and palatable. It seems probable that before long whale oil, which, in a raw state, has a most unpleasant, if not revolting, odour, will form an ingredient of cheap-food materials, for physiological experiments have demonstrated that it is easily digested. There is one point, however, which should be settled before this prepared oil comes into general use as a food-stuff: it appears that a small percentage of nickel is found in oil hardened by the new process, and the question arises whether this is in any way detrimental. It has been shown that this amount of nickel is very much less than that contained in many food-stuffs prepared in nickel vessels, and the probability is that the minute proportion is negligible.

### NEW ASSISTANT SECRETARY AT CARDIFF.

Mr. Hedley Lucas has been appointed assistant secretary at King Edward VII.'s Hospital, Cardiff. For five years he was clerk to the superintendent at Monsall Hospital, Manchester, and for the last two and a half years he has been chief clerk in the registration office, clerk-in-charge of the out-patient department, and clerk for the medical board at the Manchester Royal Infirmary. His training has also included the following: He is a chartered secretary by examination, a matriculant of London University, and a Pitman medallist; and he has contributed to Manchester papers.

### MEXICO AND THE DRUG MARKET.

A very quiet tone continues to prevail in the drug market, but there is nothing unusual in this at the present season. Of price changes there have been few of any outstanding importance. Favourable reports continue to come to hand from the Norwegian fishing districts, and it now seems probable that the yield of cod-liver oil at the end of the season will be almost as abundant as that in the record year of 1912; as was mentioned last week, however, there is little room for further reductions in price, and possibly no great advantage would be obtained by delaying purchases. This season's oil, by the way, is of very good quality. The high price of citric acid is firmly maintained; tartaric acid has an upward tendency in price. The value of American peppermint oil still tends in an upward direction. A drug which is attracting interest at the moment is Mexican jalap, and it is not improbable that if war between the United States and Mexico is not averted the price of jalap will advance; up to the present, however, the rumours of war do not appear to have affected the price to any appreciable extent. Menthol continues very dull. There is no change in the price of cocaine, and, notwithstanding the large stocks it might not be altogether inadvisable to take advantage of the present low quotations to make necessary purchases.

### TO OUR READERS.

Contributions are specially invited from any of our readers to these columns. They should deal with topical subjects and news. They must be authenticated for the information of the Editor only. The minimum payment if published is 5s. There is no hard-and-fast rule as to space, but notes of about twenty lines in length are preferred.

